# Investigating Emotional Body Posture Recognition in Adolescents with Conduct Disorder Using Eye-Tracking Methods

**DOI:** 10.1007/s10802-021-00784-2

**Published:** 2021-02-20

**Authors:** Nayra A. Martin-Key, Erich W. Graf, Wendy J. Adams, Graeme Fairchild

**Affiliations:** 1 Department of Chemical Engineering and Biotechnology, Cambridge Centre for Neuropsychiatric Research, University of Cambridge, Cambridge, UK; 2 School of Psychology, University of Southampton, Southampton, UK; 3 Department of Psychology, University of Bath, Bath, UK

**Keywords:** Conduct disorder, Callous-unemotional traits, Body posture, Emotion recognition, Eye tracking

## Abstract

Adolescents with Conduct Disorder (CD) show deficits in recognizing facial expressions of emotion, but it is not known whether these difficulties extend to other social cues, such as emotional body postures. Moreover, in the absence of eye-tracking data, it is not known whether such deficits, if present, are due to a failure to attend to emotionally informative regions of the body. Male and female adolescents with CD and varying levels of callous-unemotional (CU) traits (*n* = 45) and age- and sex-matched typically-developing controls (*n* = 51) categorized static and dynamic emotional body postures. The emotion categorization task was paired with eye-tracking methods to investigate relationships between fixation behavior and recognition performance. Having CD was associated with impaired recognition of static and dynamic body postures and atypical fixation behavior. Furthermore, males were less likely to fixate emotionally-informative regions of the body than females. While we found no effects of CU traits on body posture recognition, the effects of CU traits on fixation behavior varied according to CD status and sex, with CD males with lower levels of CU traits showing the most atypical fixation behavior. Critically, atypical fixation behavior did not explain the body posture recognition deficits observed in CD. Our findings suggest that CD-related impairments in recognition of body postures of emotion are not due to attentional issues. Training programmes designed to ameliorate the emotion recognition difficulties associated with CD may need to incorporate a body posture component.

## Introduction

Conduct disorder (CD) is characterized by a persistent pattern of antisocial behavior that involves the violation of others’ rights or age-appropriate societal norms (American Psychiatric Association, 2013). It is one of the most common, and highly impairing, psychiatric disorders of childhood and adolescence (Erskine et al., 2016; Fairchild et al., 2019). An important personality factor when considering the aetiology of CD is callous-unemotional (CU) traits – i.e., a lack of concern for other people’s feelings, superficial affect, and a deficient sense of guilt or regret (Pardini & Frick, 2013). These traits are of potential clinical significance, particularly given the inclusion of the ‘limited prosocial emotions’ specifier to CD in the DSM-5 (American Psychiatric Association, 2013), which maps closely onto the concept of CU traits (Pardini & Frick, 2013).

Children and adolescents with CD and/or elevated CU traits are reported to show deficits in facial emotion recognition. The mechanisms underlying such difficulties, such as atypical patterns of attending to, or processing of, emotionally-salient information, are not well understood. While adolescents with clinically-diagnosed CD show impaired recognition of negative facial expressions or global emotion recognition problems (Fairchild et al., 2010; Fairchild et al., 2009; Kohls et al., 2020; Martin-Key et al., 2018; Short et al., 2016; Sully, Sonuga-Barke & Fairchild, 2015), high levels of CU traits appear to be specifically associated with difficulties in recognising distress cues in others, such as sad (Fairchild et al., 2010) or fearful facial expressions (Dadds et al., 2006; Marsh & Blair, 2008).

The latter deficit in fear recognition has been associated with a failure to attend to emotionally relevant regions of the face, such as the eyes (Dadds, El Masry, Wimalaweera & Guastella, 2008; Dadds et al., 2006). Dadds et al. (2008) reported selective deficits in fear recognition in male adolescents who were high in CU traits. Importantly, the authors used eye tracking to show that the high CU traits group fixated less on the eye region of the face than the low CU traits group. When participants were instructed to look at the eye region, the fear recognition deficit observed in adolescents with high levels of CU traits was ameliorated, suggesting that their emotion recognition deficits were driven by attentional issues.

In a recent eye-tracking study, we found that having CD and being male were independent predictors of poorer recognition of facial expressions in general, and this was true across static and dynamic facial stimuli (Martin-Key et al., 2018). Having CD and being male were also associated with reduced attention to the eyes, particularly when viewing sad, surprised, and fearful expressions. Critically, however, reduced orienting to the eye region did not account for the facial emotion recognition deficits shown by the CD group. This runs counter to the findings of Dadds et al. (2008), and suggests that overt attention-based issues are *not* responsible for the facial emotion recognition difficulties observed in CD.

In naturalistic settings, humans do not rely solely on facial expressions when identifying others’ emotional states. Other emotional channels, such as vocal tone and body posture, also play a key role in social communication. In fact, some emotions may be communicated more effectively via body posture than facial expressions. For example, aggression may be perceived as more of a direct risk when portrayed via the body (e.g., a clenched fist indicating anger) rather than an angry facial expression (de Gelder et al., 2010). Furthermore, individuals may rely more on emotional information derived from body postures than facial expressions when these are incongruent (de Gelder, 2006). Body posture and facial expressions have been described as equally informative and are readily recognized when determining the emotion being expressed (Reed, Stone, Bozova & Tanaka, 2003; Reed, Stone, Grubb & McGoldrick, 2006).

To date, only two studies have examined emotion recognition conveyed by body posture in male youth with varying levels of CU traits (Muñoz, 2009; Wolf & Centifanti, 2014). The first study showed that high levels of CU traits and high levels of aggression were independently related to difficulties in fear recognition from static body postures (Muñoz, 2009). More recently, Wolf and Centifanti (2014) found that high levels of CU traits were associated with impaired recognition of dynamic point-light depictions of angry body postures and facial expressions of pain (but not impaired fear recognition). Despite the discrepancies between these studies, collectively they suggest that both CU traits and aggression are associated with deficits in recognition of facial and body postures of emotion, and that these impairments may not be specific to fear.

The existing literature leaves a number of important questions unanswered. First, given the focus on CU traits in healthy populations in previous studies of body posture recognition, it is unclear whether the broader impairments in facial emotion recognition found in adolescents with clinically-diagnosed CD (Fairchild et al., 2009, 2010; Kohls et al., 2020; Martin-Key et al., 2018; Sully et al., 2015) extend to body posture recognition difficulties. Second, as the earlier body posture studies recruited male-only samples, it is unclear whether females with CD (or elevated CU traits) have comparable difficulties. Third, previous studies may have overestimated the effects of CU traits due to the artificial nature of the stimuli used (i.e., static stimuli, dynamic point-light displays of body postures). It is therefore important to investigate the recognition of both static and realistic dynamic portrayals of body postures of emotion. Finally, if either males or females with CD (or both groups) show deficits in recognition of emotional body postures, it is important to ascertain if these are due to attention-based issues.

To address these issues, the present study assessed the recognition of static and dynamic body postures of emotion in male and female adolescents with CD and age- and sex-matched typically-developing (TD) controls. It should be noted that the stimulus set used in the current study was restricted to just angry, fearful and neutral body postures. This was largely for pragmatic reasons – it was felt that the dynamic stimulus set created by Jessen, Obleser and Kotz (2012) was the most realistic and highest quality set of body posture stimuli available. It also covered the two emotions reported to be impaired in previous work on body posture recognition in youth with CU traits, namely angry and fearful (Muñoz, 2009; Wolf & Centifanti, 2014), as well as neutral body postures. The latter are important in terms of interpreting the findings for negatively-valenced emotions, and can help researchers to determine whether there is a global effect of CD or CU traits on body posture recognition (which would manifest as deficits across all emotion categories, including neutral) or a specific deficit for negative emotions. Unfortunately, there are no well-validated body posture stimulus sets which include high quality dynamic portrayals of the six basic emotions. Eye fixation behavior during the task was recorded to investigate whether individuals with CD show atypical fixation behavior when processing body postures and, if so, whether fixation behavior mediates deficits in body posture recognition. A secondary aim was to examine whether CU traits are associated with impaired body posture recognition and atypical fixation behavior.

We hypothesized that individuals with CD, and particularly males, would exhibit global difficulties in body posture recognition, and that these deficits would be present across both static and dynamic stimuli. In terms of fixation behavior, we predicted that individuals with CD, and particularly males with CD, would show a weaker tendency to fixate informative regions of the body. In line with previous studies, we expected that deficits in body posture recognition (e.g., Muñoz, 2009; Wolf & Centifanti, 2014) and atypical fixation behavior (Dadds et al., 2008) would be most pronounced in those with elevated CU traits. Given that this is the first study, to our knowledge, to examine recognition of, and attention to, body postures of emotion in male and female adolescents with CD and TD controls, it was not possible to make clear predictions regarding whether atypical fixation behavior mediates the relationship between CD and CU traits and body posture recognition deficits.

## Method

### Participants

One hundred and twenty-eight adolescents aged 13–18 were recruited via Youth Offending Services and pupil referral units across Southampton and Hampshire, and through mainstream schools and colleges in Southampton via mail-shots. Of these 128, five were not eligible (see inclusion criteria below) and two TD and 10 CD participants opted not to take part in the laboratory experiment. A further five CD participants did not complete the laboratory task and six TD and four CD subjects could not be successfully eye tracked due to technical difficulties. This left a final sample of 96 participants, consisting of 45 adolescents with CD (22 male) and 51 TD adolescents (26 male). All participants and the parents of those under the age of 16 provided written informed consent to participate in the study, which was approved by the University of Southampton’s Ethics Committee and the Hampshire County Council Children's Services Research Governance Committee. All of the included participants had taken part in our previous study on facial expression recognition (Martin-Key et al., 2018) and completed both tasks during the same session.

The inclusion criteria for the study were: (i) being fluent in English; (ii) being aged between 13–18 years; and (iii) having an estimated Full-Scale Intelligence Quotient (IQ) ≥ 70 (as assessed using the Wechsler Abbreviated Scale of Intelligence; Wechsler, 1999). Exclusion criteria for the study were: (i) wearing bi/tri-focal glasses or hard contact lenses, as this could affect fixation behavior recordings, and (ii) having Autism Spectrum Disorder (ASD) or Psychosis.

## Measures and Procedure

### The Schedule of Affective Disorders and Schizophrenia for School-Aged Children - Present and Lifetime version (K-SADS-PL).

The K-SADS-PL (Kaufman et al., 1997) is a semi-structured diagnostic interview based on DSM-IV criteria. It was employed to assess participants for a range of disorders including CD, Attention-Deficit/Hyperactivity Disorder (ADHD), Major Depressive Disorder (MDD), Generalized Anxiety Disorder (GAD), Psychosis, Post-Traumatic Stress Disorder (PTSD), and Alcohol and Substance Use Disorders. ASDs were evaluated using the ASD component of the unpublished DSM-5 version of the K-SADS-PL (kindly provided by Joan Kaufman). As suggested by Kaufman et al. (1997), a symptom was considered present if reported by either the participant or the parent/carer. Interviews were administered by trained post-graduate students and the inter-rater reliability of CD and other disorders in the current study was excellent (Cohen’s kappas ranged from 0.87–1.00).

### The Inventory of Callous-Unemotional Traits

The self-report version of the Inventory of Callous-Unemotional traits (ICU; Frick, 2003) is a 24-item questionnaire focusing on the affective and interpersonal components of psychopathy. Items (e.g., ‘I do not care who I hurt to get what I want’) are measured on a scale from zero (‘not at all true’) to three (‘definitely true’). Internal consistency in the present sample was good (Cronbach’s alpha = 0.82 (entire sample), 0.80 (CD group)).

### Body Posture Categorization Task

This task assessed participants’ ability to categorize dynamic and static body postures. The original stimulus set included 360 video-clips and were developed and validated by Jessen et al. (2012) with healthy adults (*N* = 16) who recognized the expressed emotions with 96.2% accuracy. The stimulus set includes angry, fearful and neutral emotional states *only* and the actors’ faces have been blurred to eliminate emotional information portrayed via facial expressions. For the purposes of our study, we selected 42 video-clips (dynamic stimuli). We then created 42 static stimuli from these dynamic sequences by extracting one highly identifiable frame from each video-clip, using Matlab 7.1.9 (TheMathWorks Inc. Natick, MA), resulting in a total of 84 trials. Stimuli were displayed on a 19-inch monitor with a screen resolution of 1,024 × 768 pixels and subtended approximately 10.5° of visual angle at a viewing distance of 60 cm.

**Fig. 1:**
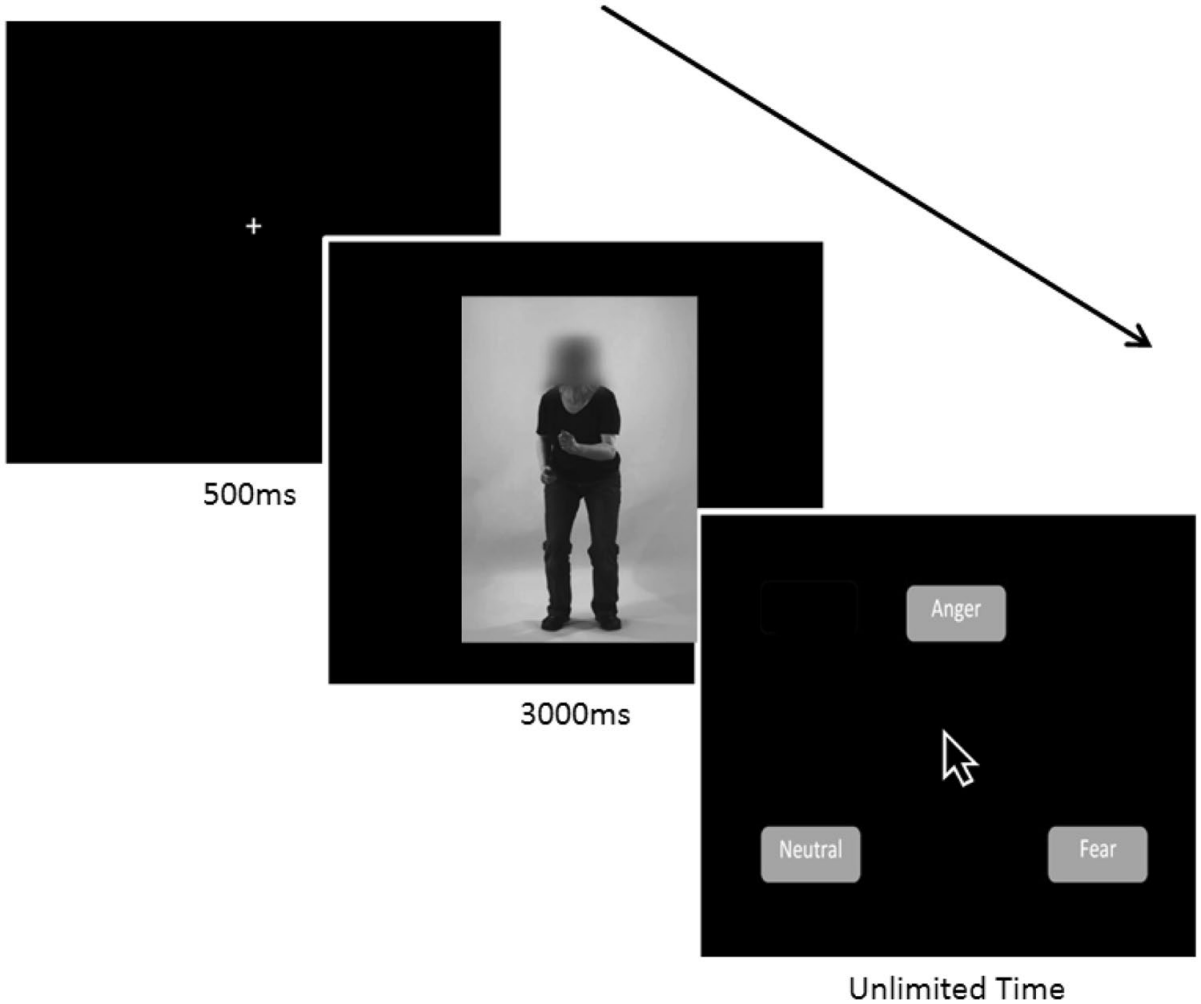
Task design and example of the body expression stimuli used in the study. Participants viewed body expressions of emotion for 3000 ms and were then asked to label the emotion. Fixation behavior data were collected in parallel, following an initial calibration procedure

Participants completed the 84 trials in two blocks of 42 randomly interleaved trials (two actors (one female, one male) x three emotions x seven repetitions x two stimulus types (dynamic, static)), taking a break between the blocks as needed. Each trial began with a 500 ms fixation cross. Following a 3000 ms stimulus presentation, participants were presented with three emotion labels (anger, fear, and neutral) and were required to select (via a mouse click) the label that best described the emotion presented in the video or static image (see Fig. 1). Participants had an unlimited time to respond, but were asked to do so as quickly and accurately as possible.

### Eye Fixation Behavior

An EyeLink 1000 eye tracker (SR Research Ltd., Canada) was used to record participants’ eye movements and fixation behavior at a monocular sampling rate of 1000 Hz and mean spatial accuracy of ~ 0.25 – 0.50°. A chin and forehead rest stabilized the head. Prior to the start of the experiment, each participant was required to fixate on nine target points on the screen as part of the calibration procedure. During the task, each trial commenced with a drift correction in order to maintain the accuracy of the calibration parameters. If drift error exceeded 1°, the calibration procedure was repeated and the trial would only commence if appropriate calibration was achieved.

## Data Analyses

### Demographic Characteristics

Participants whose parents’ professions were classed as ‘high or intermediate’ (as defined by the UK Office for National Statistics, 2010) were categorized as high socioeconomic status (SES), while those with parents in ‘routine, manual, or unemployed’ categories were classified as low SES. Given the limited range of self-reported ethnicities in the current sample, participants were classified as either Caucasian or non-Caucasian. One-way ANOVAs were employed to assess group differences in continuous variables, while Chi-Square tests (χ^2^) were used to compare group differences in binary variables.

### Behavioral Data

Linear mixed-effects model (LMM) analyses^1^ were used to analyse the categorization accuracy data. These were conducted in R version 3.6.1 (R Core Team) using the *‘lme4′* package. We examined the effects of CD status, sex, CU traits, IQ, SES, stimulus type (dynamic *vs.* static), and the two-way interactions between these (fixed effect) predictor variables, on: (i) overall categorization accuracy, and (ii) categorization accuracy for individual emotions. Models included subject, age, and psychiatric comorbidity as random factors.

When investigating overall categorization accuracy, emotional expression (fear, anger, and neutral) was also included as a random factor, thereby providing the maximum power to assess the effects of key predictors while accounting for the variance introduced by emotional expression. We subsequently conducted separate analyses for each individual emotion. For each model, the significance of each predictor was defined by likelihood ratio tests comparing models with and without each predictor (see Appendix S1: Data Analytic Strategy in Supplementary Materials, available online, for further details). Simple effect sizes for individual predictors are quantified using Cohen’s *d* (small ≥ 0.20, medium ≥ 0.50, large ≥ 0.80; Cohen, 1988). We also report Cohen's *f*^2^ values, which can be used to quantify the degree of variance explained by a single predictor, when accounting for all other predictors in the model (see Table S2 in Supplementary Materials, available online).

### Fixation Behavior Data

It has been suggested that the arms and hands may contain diagnostic information for distinguishing emotions from body posture (Dael, Mortillaro & Scherer, 2012). Therefore, and given the characteristics of the stimuli used in the current study (i.e., anger was depicted by raising the arms, fear was presented by moving the arms towards the body, and neutral body postures included grooming movements, such as moving the arms towards the head or scratching; see Jessen & Kotz, 2011), we classified the arms as the most emotionally-relevant regions of interest (ROIs) for each of the three emotions. During the analysis phase, these regions were manually identified and tracked across frames using bespoke software. The participant’s fixation position at each 1 ms time point was compared to the location of these ROIs. We then computed *arm preference scores,* operationalized as the percentage of time spent fixating the arms in each trial. The first fixation was omitted, as fixation on the central cross was required to initiate the trial. The extent to which arm preference was predicted by CD status, sex, CU traits, IQ, SES, stimulus type (dynamic *vs.* static), and the two-way interactions between these variables was determined using LMMs. Models included subject, age, and psychiatric comorbidity as random factors.

### Relating Fixation Behavior to Categorization Accuracy

Lastly, we tested whether arm preference scores were significant predictors of body posture categorization. We also assessed whether differences in fixation behavior mediated the relationship between participant characteristics (e.g., CD status, sex, CU traits) and emotion categorization performance. To this end, we determined whether adding arm preference to the best-fitting models of emotion categorization resulted in significant improvements in the predictive power of the models, which would suggest that the associations were explained (i.e., mediated) by fixation behavior.

## Results

### Participant Characteristics

The demographic and clinical characteristics of the sample are reported in Table 1. The four groups did not differ significantly in age or ethnicity. However, the CD groups had significantly lower IQ scores than the TD groups. Furthermore, CD males were more likely to come from low SES backgrounds than TD males. Finally, males with CD had significantly higher levels of CU traits than both of the TD groups, while females with CD had higher levels of CU traits than TD females.

**Table 1:** Demographic and clinical characteristics of the sample

	TD Males^1^ (n = 26)	CD Males^2^ (n = 22)	TD Females^3^ (n = 25)	CD Females^4^ (n = 23)			
	M (SD)				F		Post-hocs^a^
Age (years)	16.22 (1.40)	15.80 (1.92)	16.40 (1.80)	16.36 (1.52)	0.60		-
IQ	104.65 (11.63)	85.64 (6.95)	100.40 (12.64)	91.91 (16.90)	11.05***		1, 3 > 2, 4
ICU	22.88 (6.08)	30.23 (7.97)	17.96 (6.47)	26.65 (9.11)	11.72***		1, 3 < 2; 3 < 4
	n (%)				χ^2^		
High SES^b^	17 (65)	5 (23)	14 (56)	10 (43)	9.60*		1 > 2
Low SES	4 (15)	11 (50)	8 (32)	8 (35)			
Caucasian	21 (81)	21 (95)	24 (96)	22 (96)	5.55		-
ADHD	-	10 (36)	-	5 (22)	2.85		-
MDD	-	5 (20)	-	4 (17)	0.20		-
Anxiety	-	1 (5)	-	4 (35)	1.88		-
Substance abuse	-	0 (0)	-	1 (4)	-		
Alcohol abuse	-	0 (0)	-	1 (4)	-		
PTSD	-	0 (0)	-	1 (4)	-		

The presence of a current psychiatric disorder was an exclusion criterion for the TD group. Key: *ADHD* Attention-Deficit/Hyperactivity disorder, *CD* Conduct Disorder, *ICU* Inventory of Callous-Unemotional traits, *IQ*, Intelligence Quotient, *MDD* Major Depressive Disorder, *PTSD* Post-Traumatic Stress Disorder, *SD* Standard Deviation, *SES S*ocioeconomic Status, *TD* Typically-Developing ^a^ numbers in post-hocs column relate to the superscripts next to the group labels; ^b^ data on SES were unavailable for around 20% of the sample

*p < 0.05; **p < 0.01; ***p < 0.001

### Overall Categorization Accuracy

**Fig. 2:**
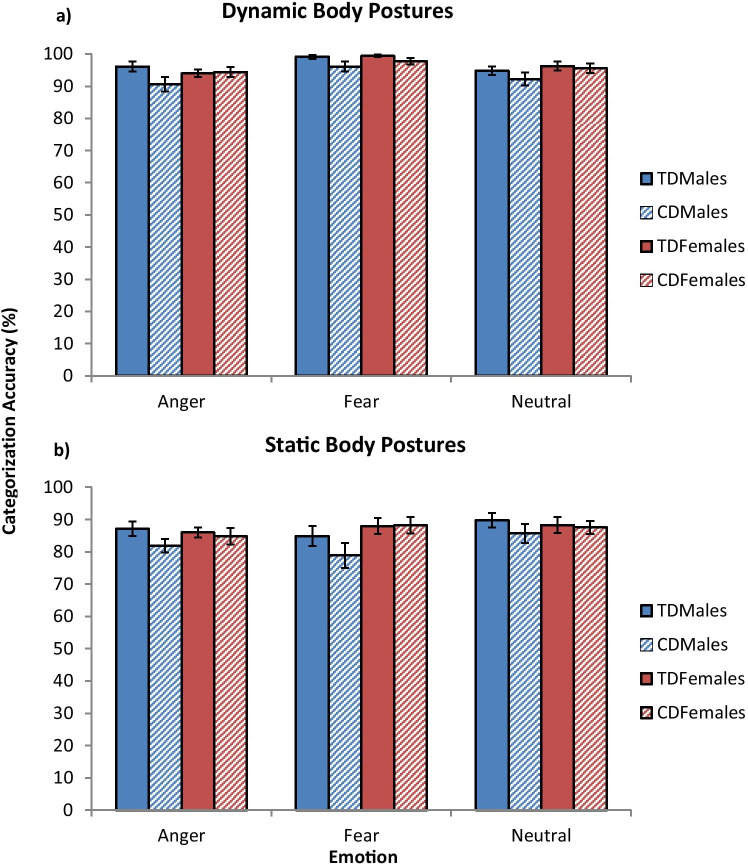
Mean emotion categorization accuracy data for each group, as a function of emotion and stimulus type. The findings obtained with dynamic stimuli are shown in panel **a**), whereas those obtained with static stimuli are presented in panel **b**). *Note:* error bars show ± standard error. CD, conduct disorder; TD, typically-developing

Emotion categorization performance, separated by group, sex, and stimulus type (static *vs.* dynamic), is presented in Fig. 2, with the results of the LMM analyses reported in Table 2. Having a diagnosis of CD was associated with significantly lower overall emotion categorization accuracy (*d* = 0.47). We also found a significant interaction between CD status and sex (*d* = 0.42). Post-hoc pairwise comparisons revealed significantly lower *overall* categorization accuracy in CD males relative to both TD males and CD females, however, these differences did not survive correction for multiple comparisons (using Bonferroni).

**Table 2:** Simplified models for body posture categorization and eye-movement behavior, across all emotions and for individual emotions

		Significant Predictors (*B*)									
		CD	Sex	CU	IQ	Stimulus type	CD*Sex	CD*CU	CD*IQ	Sex*CU	Sex*IQ
Categorisation Accuracy	All	-4.63^*^	-	-	-	-9.60^***^	4.81^*^	-	-	-	0.16^*^
	Anger	-	-	-	-	-9.12^***^	-	-	-	-	-
	Fear	-	-	-	-	-13.07^***^	-	-	-	-	-
	Neutral	-	-	-	-	-6.61^***^	-	-	-	-	-
Total Arm Preference	All	-25.90^**^	8.65^**^	0.23^*^	-0.13^*^	-8.37^***^	-	-	0.23^**^	-0.31^*^	-
	Anger	-	-	-	-	-4.29^***^	-	-	-	-	-
	Fear	-39.68^***^	9.14^*^	-	-	-9.08^***^	-	0.39^*^	0.27^*^	-0.32^*^	-
	Neutral	-11.53^**^	9.26^**^	-1.13^*^	-0.31^*^	-11.74^***^	-	0.38^*^	-	-0.37^**^	-

Key: *B,* unstandardized coefficient; *CD* Conduct Disorder, *CU* Callous-Unemotional Traits, IQ Intelligence Quotient. Total arm preference, the percentage of the overall trial time spent fixating the arm region.

*p < 0.05, **p < 0.01, ***p < 0.001 as determined by likelihood ratio tests

There was a significant interaction between sex and IQ (*d* = 0.44): IQ was marginally positively correlated with categorization accuracy in females (*r* = 0.12, *p* = 0.051) but not in males (*r* = 0.05, *p* = 0.40). In addition, categorization accuracy was higher for dynamic than static body postures of emotion (*d* = 0.65), but neither CD status, sex, nor CU traits interacted with stimulus type.

### Categorization Accuracy for Individual Emotions

Contrary to our expectations, categorization accuracy was not significantly predicted by CD status, sex, or CU traits when considering each emotion separately. Categorization accuracy was higher for dynamic than static body postures of emotion for each emotion separately (*ds* ≥ 1.44), but neither CD status, sex, nor CU traits interacted with stimulus type.

### Overall Fixation Behavior

**Fig. 3:**
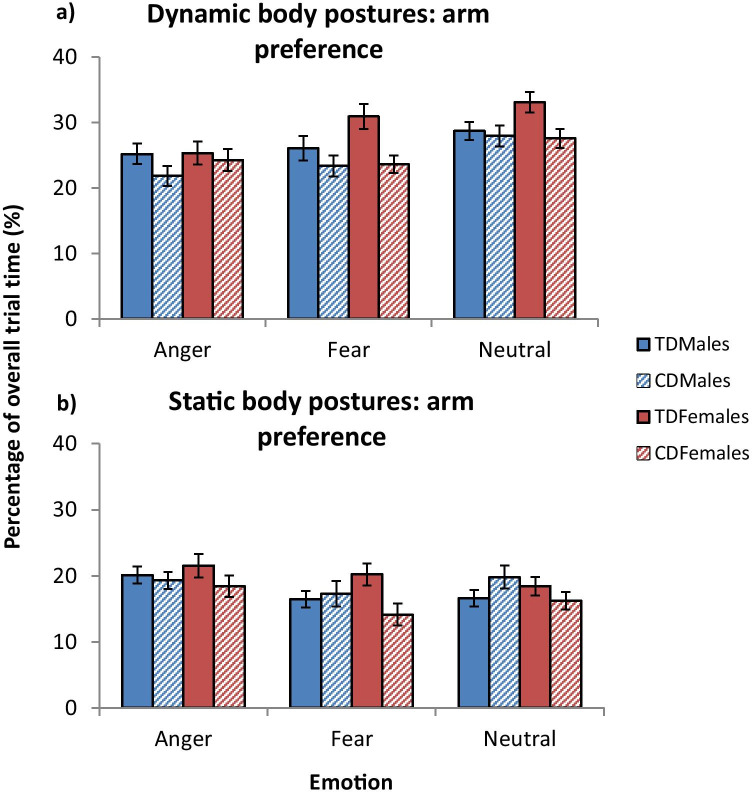
Mean fixation behavior data for each group, split by emotion and stimulus type. Arm preference scores for dynamic stimuli are shown in panel **a**), whereas those obtained with static stimuli are presented in panel **b**). *Note:* error bars show ± standard error. CD, conduct disorder; TD, typically-developing

Arm preference scores, as a function of CD status, sex, and stimulus type (static *vs.* dynamic), are shown in Fig. 3, with the results of the LMM analyses presented in Table 2. Relative to controls, participants with CD showed lower arm preference scores across all emotions (*d* = 0.68). Sex was also a significant predictor of fixation behavior: relative to females, males showed lower arm preference scores across all emotions (*d* = 0.56), but CD status did not interact with sex. We found that higher levels of CU traits (across the entire sample) predicted higher arm preference scores (*d* = 0.49) although this was qualified by an interaction between CU traits and sex (*d* = 0.52): CU traits were negatively associated with arm preference scores in females (*r* = -0.15, *p* < 0.05), while the opposite relationship was found in males (*r* = 0.15, *p* < 0.05).

Having lower IQ was associated with higher arm preference scores (*d* = 0.44) and there was a significant interaction between CD status and IQ (*d* = 0.59): IQ scores were positively (albeit weakly) associated with arm preference scores in the CD group (*r* = 0.12, *p* < 0.05), while the opposite pattern was found in the TD group (*r* = -0.21, *p* < 0.001). Finally, arm preference scores were significantly higher for dynamic than static body postures (*d* = 1.45) but stimulus type did not interact with CD status, sex, or CU traits.

### Fixation Behavior for Individual Emotions

Relative to controls, participants with CD showed lower arm preference scores when viewing fearful and neutral body postures (*ds* ≥ 0.62). Sex was also a significant predictor of fixation behavior: relative to females, males showed lower arm preference scores when viewing fearful and neutral body postures (*ds* > 0.46). Furthermore, higher CU traits were associated with lower arm preference scores when viewing neutral body postures (*d* = 0.45).

We found an interaction between CD status and CU traits for neutral body postures (*d* = 0.51): CU traits were positively associated with arm preference scores in the CD group, while the reverse pattern was found in the TD group (post-hoc correlations were non-significant, however: CD: *r* = 0.15, *p* = 0.17; TD: *r* = -0.10, *p* = 0.35). Similarly, CD status interacted with CU traits when viewing fearful body postures (*d* = 0.47): higher CU traits were associated with higher arm preference scores in the CD group (*r* = 0.23, *p* < 0.05), while the reverse pattern was found in the TD group (*r* = -0.20, *p* < 0.05).

Sex also interacted with CU traits when viewing fearful and neutral body postures (*ds* ≥ 0.42). CU traits were negatively associated with arm preference scores in females (*r* = -0.21, *p* < 0.05), but not in males (*r* = 0.11, *p* = 0.29) when viewing fearful body postures. Similarly, there was a negative association between CU traits and arm preference scores when viewing neutral body postures for females, while the reverse was found for males (post-hoc correlations were non-significant, however, males: *r* = 0.16, *p* = 0.12; females: *r* = -0.14, *p* = 0.19).

IQ was positively associated with arm preference scores when viewing neutral body postures (*d* = 0.49). CD status interacted with IQ to predict arm preferences when viewing fearful body postures (*d* = 0.52): here, lower IQ scores were associated with higher arm preference scores in TD (*r* = -0.24, *p* < 0.05) but not CD subjects (*r* = 0.17, *p* = 0.11). Finally, arm preference scores were significantly higher for dynamic than static body postures when considering each emotion separately (*ds* ≥ 1.06). Importantly, stimulus type did not interact with CD status, sex, or CU traits.

### Fixation Behavior as a Predictor of Categorization Accuracy

Next, we tested whether fixation behavior (i.e., arm preference) was a significant predictor of body posture categorization accuracy, when CD status, sex, CU traits, IQ, SES, and stimulus type (dynamic *vs.* static) were not included in the model. Pearson correlations between fixation behavior and categorization accuracy across all emotions and per individual emotion are reported in Table S3 (see Supplementary Materials, available online). These analyses revealed that higher arm preference scores were associated with higher categorization accuracy overall and each emotion considered separately (*Bs* ≥ 0.20, *ps* < 0.05, *ds* ≥ 0.33).

We then explored whether fixation behavior accounted for variations in emotion categorization when considered alongside other predictors of categorization accuracy. Because *overall* categorization performance (i.e., across emotions) was modulated by CD status, CD*sex and sex*IQ interactions, and stimulus type, we assessed whether adding arm preference to the best-fitting overall categorization model significantly improved the model’s predictive power. However, including arm preference did not improve the model’s ability to explain emotion categorization. These analyses suggest that group differences in body posture recognition are not mediated by differences in fixation behavior.

## Discussion

The present study assessed recognition of emotional body postures in male and female adolescents with Conduct Disorder (CD) and varying levels of callous-unemotional (CU) traits compared with sex- and age-matched typically-developing (TD) adolescents. We also measured eye fixation behavior during the task to investigate whether the CD participants showed atypical fixation behavior, and critically, whether this might explain group differences in body posture recognition. We found that having CD had detrimental effects on the recognition of body postures across multiple emotions. This was the case for both static and dynamic body posture stimuli, and the associated effect size was in the medium range. In line with findings from studies showing sex differences in the relationship between CD and emotion recognition/cognitive empathy (Martin-Key et al., 2018; Martin-Key, Allison & Fairchild, 2020), the detrimental effect of CD on emotional body posture recognition was larger in males than in females.

Contrary to our expectations, CU traits did not influence body posture recognition performance. This finding may appear surprising, given evidence that fearful *facial* expression recognition deficits are more pronounced in individuals with elevated CU traits (e.g., Dadds et al., 2008; Marsh & Blair, 2008). In addition, influential theories predict that CU traits should be associated with problems in identifying distress cues, such as fearful expressions (e.g., Blair, 1995; Blair, 2003). Here, we show no effect of CU traits on body posture recognition in either CD *or* TD adolescents. Our study extends previous research revealing no influence of CU traits on facial emotion recognition performance in adolescents with and without CD (Martin-Key et al., 2018; Schwenck et al., 2014, 2012; Sully et al., 2015). Here, we demonstrate a similar pattern of results in relation to emotional body postures.

When considering the fixation behavior data, we found that having CD and being male were associated with a reduced tendency to fixate the most informative arm regions of the body. These findings extend the results of our previous study on facial expression recognition (Martin-Key et al., 2018), where we found that having CD and being male were both independently related to a reduced tendency to fixate the emotionally-salient eye region of the face. Here, we demonstrate similar additive effects of CD status and sex on fixation behavior during the processing of emotional *body postures.*

Contrary to our expectations, higher levels of CU traits within our CD sample were associated with *higher* preferences towards the arm regions when viewing fearful body postures. On the other hand, higher levels of CU traits in the TD group were associated with *lower* arm preference scores when viewing fearful body postures; in other words, the effects of CU traits were only in the hypothesized direction in the TD group. CU traits also interacted with sex to predict fixation behavior: CU traits were *positively* correlated with arm preference scores in males when viewing fearful and neutral body postures. Conversely, CU traits in females were *negatively* associated with arm preference scores when viewing fearful body postures.

Taken together, these findings suggest that the effects of CU traits on attention to emotionally salient information may vary according to CD status and sex, with CD males with *lower levels of CU traits* showing the most atypical fixation behavior. While these findings may be considered surprising, they are in line with research demonstrating that antisocial youth with lower levels of CU traits are more likely to show impaired social information processing than their high CU traits counterparts (Waschbusch, Walsh, Andrade, King & Carrey, 2007). Another surprising finding relates to the association between IQ and fixation behavior, which differed according to group status (CD vs. TD). TD adolescents with lower IQs showed increased attention to the arms when viewing body postures, while arm preference scores were higher in CD participants with higher IQs. An explanation for this might be that the lower IQ TD subjects and higher IQ CD participants actually had overlapping IQs (given the difference in mean IQ between the two groups), resulting in a similar effect on fixation behavior, relative to those at the higher and lower ends of the IQ spectrum. However, further research is needed to disentangle the effects of IQ on emotion processing and fixation behavior in CD and TD populations.

Importantly, while fixation behavior was a significant predictor of body posture categorization accuracy, such that increased fixation of the arm regions was associated with higher categorization performance, our analyses did not support the idea that atypical fixation behavior (i.e., a failure to fixate the informative arm regions) mediates the relationship between CD status and body posture recognition deficits. Instead, the recognition impairments exhibited by adolescents with CD, and particularly males with the disorder, may reflect difficulties in the interpretation of emotional cues. These findings are broadly consistent with those of our earlier study (Martin-Key et al., 2018), where we found that CD-related deficits in facial expression recognition were not mediated by problems in orienting towards informative regions of the face, such as the eyes.

Considered together, our two eye-tracking studies suggest that the deficits in emotion recognition observed in adolescents with CD, and particularly males with CD, are likely to extend to emotional body postures. Furthermore, adolescents with CD, and particularly males with CD, show problems in attending to emotionally-informative regions of the face and body when processing emotional cues. We therefore propose that a failure to detect emotionally-salient information, irrespective of whether this is conveyed via facial expressions or body postures, may result in both impaired recognition of others’ emotional states *and* atypical fixation behavior, particularly in males with the disorder.

### Strengths and Limitations

To our knowledge, this is the first study to examine recognition of, and attention to, static and dynamic body postures of emotion in male and female adolescents with CD and sex-matched typically-developing controls. The use of dynamic and whole-body stimuli increased the ecological validity of this study relative to previous studies which only used static stimuli, and the use of eye-tracking methods enabled us to test whether atypical fixation behavior mediated the link between CD status and body posture recognition deficits. Furthermore, the CD and typically-developing groups were well-characterized from a clinical perspective, using highly reliable diagnostic measures (with excellent inter-rater reliability) and multiple informants.

Despite these strengths, this study had several limitations. First, due to ceiling effects and a moderate sample size, we were only able to detect group differences in overall emotion recognition, but were unable to demonstrate group differences for *individual* emotions. Similarly, the statistical power of the study to detect interactions may have been low – although it should be noted that the significant interaction terms all had medium effect sizes. Our ceiling effects for categorization accuracy of dynamic expressions may reflect the challenges of creating a naturalistic, parametrically-varying set of body-expressed emotional stimuli. In each video sequence, actors began from a neutral posture and finished with a ‘full emotion’ posture. Studies of *facial* emotion processing have often employed image-processing techniques such as morphing to create intermediate expressions of emotion, in contrast to the majority of investigations using full body poses. While it would be interesting to use body posture stimuli expressing varying intensities of emotion as has been done for facial expressions of emotion (Adams, Gray, Garner & Graf, 2010), it is important to note that behavioral studies in adults (e.g., Atkinson, Dittrich, Gemmell & Young, 2004; Coulson, 2004) and studies that have tried to develop automated algorithms for emotion recognition (e.g., Tan & Nareyek, 2009) have both demonstrated the complexity of emotional body posture recognition.

A second limitation was that the Jessen et al. (2012) stimulus set was developed to depict three affective states: angry, fearful, and neutral. Relative to the facial expression recognition tasks commonly used in the literature, where individuals are required to recognize/distinguish between six primary emotions, the use of just three emotional body postures is likely to have made the task less challenging and less sensitive. Nevertheless, we note that previous studies on body posture recognition also used a restricted set of emotions (Munoz, 2009) and one used dynamic point-light displays rather than realistic videos of actors portraying body postures (Wolf & Centifanti, 2014). In future research, it would be interesting to investigate recognition of additional emotions (particularly positive emotions). In addition, angry dynamic stimuli contained slightly lower motion content relative to fearful and neutral dynamic stimuli (Jessen et al., 2012), which may have had an impact on fixation behavior.

Finally, we note that the emotionally-salient regions (i.e., the arm ROIs) were selected by the researchers, based on their own evaluation of the emotion-relevant aspects of the stimulus (e.g., that the arm region would be most informative because anger is conveyed by a clenched fist). In future studies, ratings obtained from independent samples could be used to define emotionally informative regions of the body. Related to this, the use of alternative eye movement variables, such as fixation count and saccade length, may have yielded different conclusions. Finally, CU traits were measured using the self-report version of the Inventory of Callous-Unemotional traits, which may be influenced by social desirability effects.

## Conclusion

In the first study to investigate recognition of emotional body postures in youth with CD and varying levels of CU traits, we found that CD was associated with deficits in body posture recognition across multiple emotions (including neutral). As having CD and being male were independently linked to poorer body posture recognition, males with CD showed the greatest impairments on the task. Eye tracking data collected in parallel showed that having CD and being male were associated with a reduced tendency to fixate the most informative regions of the body. However, fixation behavior did not mediate the relationship between CD and body posture recognition impairments. These findings provide further evidence that males with CD show more global deficits in emotion recognition than their female counterparts and therefore may require more extensive emotion training intervention programs.

## Supplementary Information

Below is the link to the electronic supplementary material.

Supplementary file1 (DOCX 26.5 KB)

## Footnotes

1 This approach was chosen over the traditional ANOVA approach as it provides a principled way to include both continuous and categorical variables, whilst modelling individual subject‐ and stimulus‐related sources of variation. By more precisely modelling random variation, power to detect effects of interest is increased, without inflating the probability of Type II errors (see Baayen, Davidson & Bates, 2008; Bates, Kliegl, Vasishth & Baayen, 2015).
