# Supplementary material for: Investigating Emotional Body Posture Recognition in Adolescents with Conduct Disorder Using Eye-Tracking Methods
Source: Res Child Adolesc Psychopathol. 2021 Feb 20;49(7):849–60. doi: 10.1007/s10802-021-00784-2 (PMC8154802; doi:10.1007/s10802-021-00784-2)
Supplement: Supplementary file 1 — Supplementary file1 (DOCX 26.5 KB) [file 10802_2021_784_MOESM1_ESM.docx]

**Online supporting information for Martin-Key et al. - *Investigating emotional body posture recognition in adolescents with Conduct Disorder using eye-tracking methods***

**Appendix S1. Data Analytic Strategy:**

To create parsimonious models of the data, we followed the approach suggested by Bates, Kliegl, Vasishth, and Baayen (2015), in which the initial model includes as many random and fixed effects as are supportable by the data. This was followed by a stepwise backward elimination process to remove factors that did not significantly improve the model’s fit to the data. Models included subject, ADHD, mood, anxiety, and age as random (intercept) factors (note that PTSD, alcohol, and substance abuse were present in one subject only, and thus these variables are subsumed by the subject factor). In addition, for analyses collapsed across emotional expressions, emotion was also included as a random factor. CD, sex, CU traits, IQ, SES, and stimulus type (dynamic *vs.* static) were included as fixed effects, in addition to all two-way interactions between these variables.

Models were evaluated using the ‘*lme4*’ package in R. After fitting the most complex model, fixed factors that did not significantly improve to the model were iteratively removed. The contribution of each candidate factor was evaluated using a likelihood ratio test to compare the more complex model with a reduced (nested) model excluding the candidate factor. The model was simplified following a non-significant decrease in goodness of fit (*p* > .05); we note that inclusion/exclusion based on coefficient *t*-tests is not advised in mixed-effects models (Baayen, Davidson & Bates, 2008; Bates et al., 2015). Because IQ and SES differed significantly between participant groups, we followed the conservative approach of including these (potentially confounding) factors in the final models, even when their effects were non-significant.

**Table S1.** *Full models for body posture categorization and eye-movement behavior, across all emotions and for individual emotions.*

|  | | Significant Predictors (*B*) | | | | | | | | | | |
| --- | --- | --- | --- | --- | --- | --- | --- | --- | --- | --- | --- | --- |
|  | | CD | Sex | CU | IQ | SES | Stimulus type | CD*Sex | CD*CU | CD*IQ | Sex*CU | Sex*IQ |
| Categorization Accuracy | All | -4.63^*^ | - | - | -.09 | -.34 | -9.60^***^ | 4.81^*^ | - | - | - | .16^*^ |
|  | Anger | - | - | - | .04 | -1.56 | -9.12^***^ | - | - | - | - | - |
|  | Fear | - | - | - | .02 | -2.21 | -13.07^***^ | - | - | - | - | - |
|  | Neutral | - | - | - | .06 | .75 | -6.61^***^ | - | - | - | - | - |
| Arm Preference | All | -25.90^**^ | 8.65^**^ | .23^*^ | -.13^*^ | 1.09 | -8.37^***^ | - | - | .23^**^ | -.31^*^ | - |
|  | Anger | - | - | - | .00 | 1.22 | -4.29^***^ | - | - | - | - | - |
|  | Fear | -39.68^***^ | 9.14^*^ | - | -.13 | 1.81 | -9.08^***^ | - | .39^*^ | .27^*^ | -.32^*^ | - |
|  | Neutral | -11.53^**^ | 9.26^**^ | -1.13^*^ | -.31^*^ | .21 | -11.74^***^ | - | .38^*^ | - | -.37^**^ | - |

Key: *B,* unstandardized coefficient; CD, Conduct Disorder; CU, callous-unemotional traits; IQ, intelligence quotient; SES, socioeconomic status; arm preference, the percentage of trial time spent fixating the arm region. **p* < .05, ***p* < .01, ****p* < .001 as determined by likelihood ratio tests.

**Effect size measures**

Within the manuscript, we have reported simple effect sizes, as quantified using Cohen’s *d* (Cohen, 1988). This measure can be used, for example, to understand the difference in categorization performance between those with and without a CD diagnosis. However, in the context of multi-predictor models, we may wish to understand the contribution of CD status in predicting categorization performance, given that other variables such as IQ might account for some of the differences between these two groups. In other words, we might wish to quantify the unique contribution of CD status, after accounting for variability in emotion categorization that is associated with variables such as IQ, SES, and sex. We can use Cohen’s f-squared (*f^2^*) to quantify the effect size that is associated with a single predictor (or subset of predictors) within the context of these other variables (see Selya et al., 2012). Note that the interpretation of the magnitude of Cohen’s *f^2^* differs from that of Cohen’s *d*. According to Cohen (1988), *f^2^* ≥ .02, *f^2^* ≥ .15, and *f^2^* ≥ .35 represent small, medium, and large effect sizes, respectively. Table S2 shown below provides this ‘contextual’ effect size measure for key predictors (CD, sex, CU traits, and their interactions) within models of emotion categorization and eye movement behavior.

**Table S2.** *Effect sizes (expressed as Cohen’s f-squared) associated with key predictor variables, in the context of multi-predictor models.*

|  | | Cohen’s *f^2^* | | | | | | | |
| --- | --- | --- | --- | --- | --- | --- | --- | --- | --- |
|  | | CD | Sex | CU | CD*Sex | CD*CU | CD*IQ | Sex*CU | Sex*IQ |
| Categorization Accuracy | All | .11 | - | - | .09 | - | - | - | .09 |
|  | Anger | - | - | - | - | - | - | - | - |
|  | Fear | - | - | - | - | - | - | - | - |
|  | Neutral | - | - | - | - | - | - | - | - |
| Arm Preference | All | .15 | .13 | .11 | - | - | .13 | .12 | - |
|  | Anger | - | - | - | - | - | - | - | - |
|  | Fear | .32 | .20 | - | - | .20 | .21 | .18 | - |
|  | Neutral | .23 | .21 | .16 | - | .19 | - | .21 | - |

Key: CD, Conduct Disorder; Cohen’s *f^2^*, effect size measure in the context of multi-predictor models (where *f^2^* ≥ .02, *f^2^* ≥ .15, and *f^2^* ≥ .35 represent small, medium, and large effect sizes, respectively); CU, callous-unemotional traits; IQ, intelligence quotient; arm preference, the percentage of trial time spent fixating the arm region.

**Table S3.** *Correlations between arm preference scores and categorization accuracy, across all emotions and for individual emotions.*

|  | Categorization Accuracy | | | |
| --- | --- | --- | --- | --- |
| Arm Preference | All | Anger | Fear | Neutral |
|  | .17** | .13 | .19** | .16* |

Key: **p* < .05, ***p* < .01

**References:**

Baayen, R., Davidson, D., & Bates, D. (2008). Mixed-effects modeling with crossed random effects for subjects and items. *Journal of Memory and Language, 59* (Special Issue: Emerging Data Analysis), 390-412. doi:10.1016/j.jml.2007.12.005

Bates, D., Kliegl, R., Vasishth, S., & Baayen, H. (2015). *Parsimonious Mixed Models.* ArXiv e-prints.

Cohen, J. (1988). *Statistical power analysis for the behavioral sciences.* Hillsdale, N.J.: L. Erlbaum Associates.

Selya, A., Rose, J., Dierker, L., Hedeker, D., & Mermelstein, R. (2012). A practical guide to calculating Cohen's f^2^, a measure of local effect size, from PROC MIXED. *Frontiers in Psychology, 3*(4), doi:10.3389/fpsyg.2012.00111
